# SARS-CoV-2 epitope-specific T cells: Immunity response feature, TCR repertoire characteristics and cross-reactivity

**DOI:** 10.3389/fimmu.2023.1146196

**Published:** 2023-03-10

**Authors:** Gang Yang, Junxiang Wang, Ping Sun, Jian Qin, Xiaoyun Yang, Daxiang Chen, Yunhui Zhang, Nanshan Zhong, Zhongfang Wang

**Affiliations:** ^1^ Faculty of Life Science and Technology, Kunming University of Science and Technology, Kunming, China; ^2^ Guangzhou Laboratory, Guangzhou, China; ^3^ Department of Pulmonary and Critical Care Medicine, The First People’s Hospital of Yunnan Province, Kunming, China; ^4^ State Key Laboratory of Respiratory Disease & National Clinical Research Center for Respiratory Disease, Guangzhou Institute of Respiratory Health, The First Affiliated Hospital of Guangzhou Medical University, Guangzhou Medical University, Guangzhou, China

**Keywords:** SARS-CoV-2, T cell immunity, epitopes, TCR repertoire, cross-reactivity

## Abstract

The devastating COVID-19 pandemic caused by SARS-CoV-2 and multiple variants or subvariants remains an ongoing global challenge. SARS-CoV-2-specific T cell responses play a critical role in early virus clearance, disease severity control, limiting the viral transmission and underpinning COVID-19 vaccine efficacy. Studies estimated broad and robust T cell responses in each individual recognized at least 30 to 40 SARS-CoV-2 antigen epitopes and associated with COVID-19 clinical outcome. Several key immunodominant viral proteome epitopes, including S protein- and non-S protein-derived epitopes, may primarily induce potent and long-lasting antiviral protective effects. In this review, we summarized the immune response features of immunodominant epitope-specific T cells targeting different SRAS-CoV-2 proteome structures after infection and vaccination, including abundance, magnitude, frequency, phenotypic features and response kinetics. Further, we analyzed the epitopes immunodominance hierarchy in combination with multiple epitope-specific T cell attributes and TCR repertoires characteristics, and discussed the significant implications of cross-reactive T cells toward HCoVs, SRAS-CoV-2 and variants of concern, especially Omicron. This review may be essential for mapping the landscape of T cell responses toward SARS-CoV-2 and optimizing the current vaccine strategy.

## 1 Introduction

Severe acute respiratory syndrome coronavirus 2 (SARS-CoV-2) and multiple emerging variants or subvariants have caused a persistent global coronavirus disease 2019 (COVID-19) pandemic over the past three years, particularly devastating to elderly patients with underlying diseases, and resulting in more than 6 million COVID-19-related deaths (1–3). A characteristic feature of COVID-19 is that the clinical manifestations vary widely in the population, with some individuals presenting as asymptomatic or mildly infected, while others become severe respiratory failure or even death, but the mechanisms underlying this major difference have not been fully elucidated (4, 5). Recent evidence demonstrated that asymptomatic COVID-19 individuals have highly multifunctional SARS-CoV-2-specific T cell responses (6, 7), and coordinated and robust CD4+ and CD8+ T cell immunity are associated with milder disease (8). It is therefore obvious that T cells mediated protective immunity plays a critical role in controlling SARS-CoV-2 infection, which relies on CD4+ T cells to secrete a series of cytokines to support B cell-mediated antibody responses and promote the function of innate immune cells, while CD8+ T cells mediate direct antiviral function through killing infected host cells by a variety of mechanisms (9, 10).

Activated T cells targeting different SARS-CoV-2 antigen proteins were reported to be detected in up to 70% of acute and convalescent COVID-19 individuals (11, 12). The magnitude of CD4+ and CD8+ T cell response is closely related to almost all SARS-CoV-2 proteins, but a few virus antigens [spike, nucleocapsid, Membrane, non-structural proteins (nsp) 3, nsp4, nsp12, nsp13, open reading frame (ORF)3a, ORF8] cover 80% of T cell responses, suggesting a distinct immunodominant pattern (13). However, previous studies involving T cell responses toward total viral proteins rather than single epitopes after natural infection or vaccination may underestimate or even obscure the T cell immune characteristics (14). Recent studies of identification and characterization of SARS-CoV-2-specific T cell epitopes covered the most prevalent human leukocyte antigen (HLA) allelic variants worldwide (14–38), reporting that CD4+ and CD8+ T cell responses in each individual recognize at least 30 to 40 SARS-CoV-2 antigen epitopes (13), and different epitopes-specific T cell responses associate with COVID-19 disease severity and clinical outcome (23). These studies mainly used multiple methodologies such as enzyme-linked immunospot (ELISpot), intracellular cytokine staining (ICS), activation induced marker (AIM) and multimer staining for epitope identification, and single-cell RNA or T cell receptor (TCR) sequencing analysis for more detailed epitope-specific T cell information. Those critical studies provided the outstanding opportunity for a deeper understanding of SARS-CoV-2 immunodominant epitopes-specific T cells with the responses abundance, magnitude, frequency, phenotypic feature, kinetics, biological functions, TCR repertoire characteristics and immunodominance hierarchy (**Table 1**), and were thus focused and included in this review, while the literature only involving epitope identification and validation without more comprehensive and in-depth analysis was not the focus.

**Table 1:** Summary of SARS-CoV-2 immunodominant epitopes information.

Epitope Abbreviation	Peptide Source	Sequence	Known HLA restriction	Classification	Conservation (to Omicron)	Detailed information of epitope-specific T cells					Reference
						Frequency or Magnitude	phenotype in convalescents/vaccinees	TCR reportoiese	Infection or Vaccination	Kinetics following vaccination	
N105-113	Nucleocapsid	SPRWYFYYL	HLA-B*07:02	CD8	Conservative	6.88×10^-4^ in COVID-19 patients; 3.00×10^-5^ in unexposed individuals	T_cm_	Highly diverse; Bias use of the TRBV27 and longer CDR3β loops	Infection	/	(15, 16, 19, 25, 39, 40)
N322-330	Nucleocapsid	MEVTPSGTWL	HLA-B*40:01; HLA-B*44:03	CD8	Conservative	3.6×10^-5^ in early convalescent individuals; 1×10^-5^ in late convalescent individuals	Not mentioned	TRBV27/TRBJ1-4	Infection	/	(16, 20)
N361-369	Nucleocapsid	KTFPPTEPK	HLA-A11:01; HLA-B44:01; HLA-A03:01	CD8	Conservative	elicit T cell responses in 75% of COVID-19 convalescent individuals	Not mentioned	Public CDR3β motif ASSRAGTGYNEQF and ASSPSVYFEVSGANVLT	Infection	/	(20, 21, 35, 41)
S269-277	Spike	YLQPRTFLL	HLA-A*02:01	CD8	Conservative	1.65×10^-6^ in healthy individuals; 1.44×10^-5^ in COVID-19 acute and 1.28×10^-5^ in convalescent patients	T_naive_ and T_scm_ dominant naïve memory T cell	①TRAV12-1/TRAJ43, with public CVVNXXDMRF motif ②TRAV12-2/TRAJ30, with public CAVNXDDKIIF motif ③TRBV7-9	Infection and vaccination	Three epitopes specific CD8+T cells elicited by BNT162b2 peak frequency of ~3.6×10^-4^ and maintaining full function for at least 80-120 days	(17, 20, 25, 37, 42)
S1208–1216	Spike	QYIKWPWYI	HLA-A*24:02	CD8	Conservative	7.71×10^-5^ in convalescent patients; 9.50×10^-6^ in healthy individuals	diverse T_cm_, T_scm_, T_emra_ and T_naive_	Highly diverse	Infection and vaccination		(18, 20, 22, 25)
S448–456	Spike	NYNYLYRLF	HLA-A*24:02	CD8	Conservative	6.3 ×10^-5^ in convalescent patients; 8.44 ×10^-6^ in healthy individuals	diverse T_cm_, T_scm_, T_emra_ and T_naive_	TRBV2/TRBJ2-7; with public CDR3β motif XXXGYEQYF	Infection and vaccination		(18, 22, 25)
S751–767	Spike	NLLLQYGS FCTQLNRAL	HLA-DRB1*15:01	CD4	N440K mutation	1.36×10^-4^ in early convalescent patient; 3.8×10^-5^ in the late convalescent phase	T_cm_ and cTfh	bias use of TRVB24-1, TRVB20-1 and TRBV6-1, with a highly public CDR3β motif CSARRGTEAFF	vaccination	①Two epitopes specific CD4+ T cells in peripheral blood occur as early as day 7 after primary BNT162b2 vaccination and peak at days 7-14 after the second dose, maintaining for at least 200 days. ②DP04/S167-specific Tfh cell responses in lymph nodes maintain at high frequency >170-200 days	(27)
S167-180	Spike	YVSQPFLM	HLA-DPB1*04	CD4	Conservative	Not mentioned	PD-1+CXCR5+ Tfh and CD45RO+CCR7- cTfh	TRAV35,with CDR3α motif CA[G/A/V]XNYGGSQGNLIF	vaccination		(26)
M198–206	Membrane	RYRIGNYKL	HLA-A*24:02	CD8	Conservative	elicit T cell responses in 88.1% of COVID-19 convalescent individuals	CCR7-CD45RA- effector memory in moderate patients	①TRBV6-4/TRBJ1-2, with public CDR3α motif CAVXYNQGGKLIF ②TRAV1-2/TRAJ23, with CDR3β motif CASSDSGXDGYTF	Infection	/	(38)
ORF1ab 1637-1646	ORF1ab	TTDPSFLGRY	HLA-A*01:01	CD8	Conservative	reaching up to 7%-25% of the total CD8+ T cells in COVID-19 patients	Not mentioned	Highly diverse; Bias use of the TRBV27 gene fragment	Infection	/	(20, 29, 33)
ORF1ab 3886-3894	ORF1ab	KLWAQCVQL	HLA-A*02:01	CD8	Not defined	elicit T cell responses in 88.9% of COVID-19 patient	Not mentioned	TRAV38-2/DV8	Infection	/	(36)
ORF3a 139-147	ORF3a	LLYDANYFL	HLA-A*02:01	CD8	Conservative	elicit T cell responses in 88.9% of COVID-19 patient	T_cm_ and T_em1_	TRAV8-1	Infection	/	(16, 36)

Epitope conservation was achieved by sequence alignment between the original SARS-CoV-2 strain and Omicron conducted by National Center for Biotechnology Information (NCBI, https://www.ncbi.nlm.nih.gov/) and China National Center For Bioinformation (CNCB, https://ngdc.cncb.ac.cn/ncov/).

Thus far, hundreds of COVID-19 vaccines are known to be under development or have been approved to fight the pandemic. Some of these vaccines have proven to effectively reduce disease severity and mortality, suggesting a central role for vaccine-induced T cell immunity in protecting against severe tissue damage (43, 44). However, the continuous emergence of SARS-CoV-2 variants and subvariants poses a great challenge to current COVID-19 vaccine strategies (45). Those vaccines primarily focus on the SARS-CoV-2 spike glycoprotein that is highly immunogenic but susceptible to mutations in viral variants, resulting in greatly compromised protection from neutralizing antibody responses (46). Fortunately, the SARS-CoV-2 immunodominant epitope regions recognized by T cells and B cells have only minimal overlap, and awareness of this is particularly critical in vaccine design (13, 47). Hence, future vaccine strategies should cover more conserved immunodominant T cell epitopes, which are less affected by mutations and thus can induce broader and more efficient cross-protection against future SARS-CoV-2 variants and subvariants (48). Considering these challenges, this review focuses on characterizing the SARS-CoV-2 epitopes-specific T cells response feature and analyzing their TCR repertoire characteristics and cross-reactivity, which are essential for mapping the landscape of T cell responses mediated by SARS-CoV-2 and optimizing the current vaccine strategy.

## 2 SARS-CoV-2 structure and its T cell epitope

**Figure 1:**
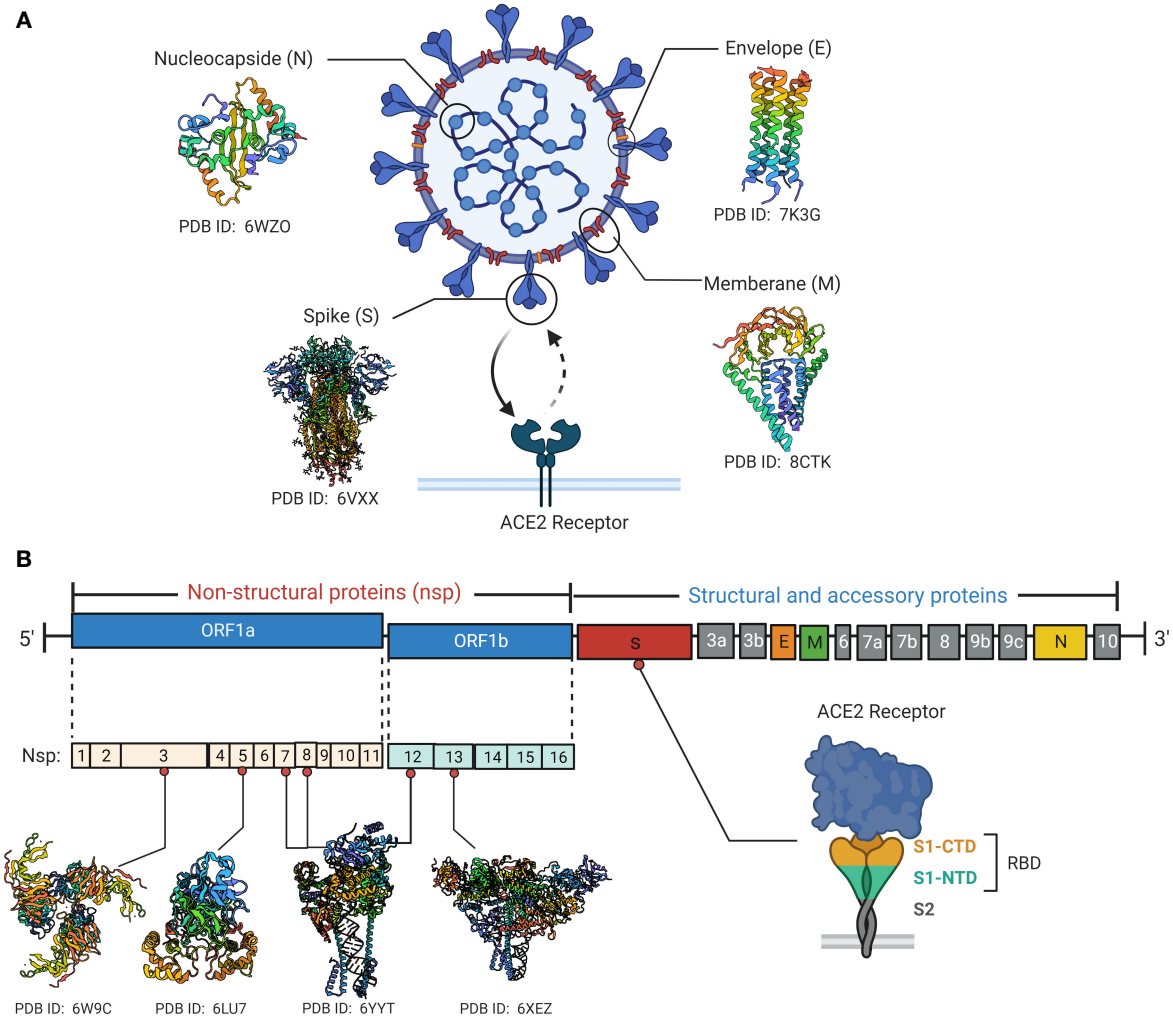
SARS-CoV-2 viral protein structure and genomic organization. **(A)** Schematic diagrams of the structural characteristics of SARS-CoV-2 as well as the crystal structures of key structural proteins obtained from RCSB protein data bank (PDB). The spike protein binds to the cell surface ACE2 receptor through the RBD. **(B)** Diagrammatic representation of SARS-CoV-2 genome contains 12 open reading frames (ORF) and encodes 16 non-structural proteins (nsp1-16), 9 accessory proteins (ORF3a-10) and 4 structural proteins. Crystal structures of Some key non-structural proteins are presented as well. Created by BioRender.com.

SARS-CoV-2 is a positive-sense RNA virus with large genome approximately of 29,903 nucleotides in length (49). The SARS-CoV-2 genome consists of 12 open reading frames (ORF) and encodes 16 non-structural proteins (NSP1-16), 9 accessory proteins (ORF3a-10) and 4 structural proteins including spike (S), envelope (E), membrane (M) and nucleocapsid (N) (**Figure 1**) (50). The M, S and E proteins on the surface of SARS-CoV-2 are embedded in the lipid bilayer membrane, while the internal N protein encapsulates viral RNA. Specifically, the S glycoprotein, composed of the S1 subunit and S2 subunit, is the main structure that mediates virus entry into host cells. It recognizes and binds to the cell surface receptor angiotensin-converting enzyme 2 (ACE2) through the receptor binding domain (RBD) to mediate membrane fusion reaction (51). The M protein can interact with multiple viral proteins and is a critical structural component for coronavirus assembly and formation (52). The E protein is the smallest structural protein and plays multiple roles in SARS-CoV-2 pathogenesis, virus packaging and release (53). The N protein directly binds to the viral RNA genome and assembles it into a ribonucleoprotein complex by synergizing with the M protein (54). In addition to structural protein components, 16 non-structural proteins remain essential in the life replication cycle of SARS-CoV-2. These nsps include various important enzymes and transcription factors such as viral protease nsp5, RNA-dependent RNA polymerase (RdRp) nsp12, helicase nsp13 and so forth (55). The 3’ end of the SARS-CoV-2 genome also encodes 9 accessory proteins including ORF3a, ORF3b, ORF6, ORF7a, ORF7b, ORF8, ORF9b, ORF9c and ORF10. The functions of these accessory proteins are not fully understood and may indirectly regulate many viral biological processes (51).

The epitope is a critical region for host immune cells to recognize a specific pathogen, also known as antigenic determinant (56). The epitopes recognized by TCR are called T cell epitopes, which are mainly peptides derived from protein degradation. T cells recognize only peptide epitopes that bind to specific class I or class II major histocompatibility complex MHC molecules, also known as MHC restriction (57). MHC class I restricted epitopes are usually 8 to 10 amino acid residues and are presented to the surface of CD8+ T cells for recognition, while class II restricted epitopes are generally 13 to 17 residues and recognized by CD4+ T cells (58).

SARS-CoV-2 genome is composed of a large 30-kb mRNA fragment and encodes various structural and non-structural proteins, thus no doubt that it has numerous T cell epitopes. Over 2000 SARS-CoV-2-derived T cell epitopes have been identified in a considerable number of previous studies, covering almost all viral proteomic structures (59, 60). However, not every SARS-CoV-2 protein epitope has the same immunodominance hierarchy. The immunodominance of a specific SARS-CoV-2 epitope usually refers to the recognition frequency and magnitude by immune cells in a given HLA-type individual or population (27, 28, 59). The epitope immunodominance is determined by multiple factors, including HLA binding affinity, TCR recognition of epitope peptide-HLA complex, and naïve T cell precursor frequency (61, 62). To date, numerous immunodominant and immunodominant epitopes have been identified not only in SARS-CoV-2 structural protein region, but also in the non-structural and accessory protein regions that are not covered by current vaccination strategies.

## 3 Detection methods of SARS-CoV-2 specific T cell

With the advancement of immunology-related basic research, the detection technologies for antigen-specific T lymphocytes are also rapidly developing and improving. ELISpot and ICS are classical techniques widely used to detect and enumerate activated antigen-specific T cells secreting cytokines such as IFN-γ or TNF-α (63, 64). Both assays combined with SARS-CoV-2 overlapping peptide pools or single epitope stimulation of peripheral blood mononuclear cells (PBMCs) or tissue samples are widely used in the detecting and identifying SARS-CoV-2 specific T cells, with the advantage of high sensitivity (17, 22, 28, 65, 66). However, these assays can only measure functional T cells with cytokine secretion, which may underestimate the number of antigen-specific T cells, and cannot isolate live cells for detailed molecular analysis (67, 68). Based on some activation-related clusters of differentiation (CD) on the T cells surface which is significantly upregulated after *in vitro* stimulation, the detection technology AIM assay (57) was developed. This assays play an important role in determining the T cell responses targeting SARS-CoV-2 that allows to detect more types of virus-specific T cell lineages and isolate live cells for multi-parameter multi-information analysis (67). In studies using AIM assay to detect SARS-CoV-2-specific T cells, there is wide variation in the activation markers chosen, with commonly used markers including CD154 (CD40L)+CD137+ OX40+ for CD4+ T cells and CD69+CD137+ for CD8+ T cells (11, 13, 27, 69). However, cell activation-based functional assays fail to detect non-functional viral specific T cells, and the cellular phenotype is prone to change under *in vitro* stimulation (31).

After nearly 25 years of development, the peptide-loaded major histocompatibility complex (pMHC) tetramer technology has become a key tool for the analysis of antigen-specific T cells, allowing direct *in vitro* detection of SARS-CoV-2 epitope-specific T cells and thus detailed exploration of the T cell response feature induced by nature infection or COVID-19 vaccine (20, 22, 27, 70, 71). Tetramer technology utilizes the biotin-streptavidin system to multimerize pMHC complexes, greatly improving the avidity and stability between pMHC and TCRs for more easily detecting or sorting for single epitope-specific T cell by flow cytometry (67). Furthermore, tetramer-associated magnetic enrichment (TAME) could be furtherly used to detect some rare SARS-CoV-2 epitope-specific T cell populations (16, 18, 32). Notably, the development of MHC class II tetramers is more difficult than the easier production of MHC class I tetramers. So, the analysis of epitope-specific CD4+ T cells is relatively limited compared to a large amount of data on epitope-specific CD8+ T cells (67, 72). Recently, Mallajosyula and his colleagues developed an improved multimeric T cell staining reagent, spheromer, that each can display 12 pMHC complexes, and reported that the staining and capture efficiency of SARS-CoV-2 epitope-specific T cells are higher than tetramer (23).

The advent of next-generation in-depth sequencing has facilitated for more detailed downstream functional and molecular characterization of single immune cell. Generally, bulk RNA sequencing for TCR repertoires can only tell the usage frequency of TCR alpha and beta chain, but could not correspond to the paring and the individual functionality. single-cell RNA sequencing (scRNA-seq) can be used to uncover the functional diversity and dissect the heterogeneity of different epitope-specific T cells based on tetramer or pentamer single cell sorted technique (73), while single-cell T cell receptor sequencing (scTCR-seq) can effectively obtain the paired TCRαβ sequences and the diversity information of clonal population, including clonotype, clonal expansion and clone functionality (25). In general, it is foreseeable that more detection, isolation and analysis techniques for antigen- and epitope-specific T cells will continue to emerge in the future, thereby clearing technical hurdles and facilitating the comprehensive and in-depth research on cellular immunity toward multiple pathogens, including SARS-CoV-2.

## 4 SARS-CoV-2 epitope-specific T cell after natural infection

### 4.1 Nucleocapsid-derived epitope-specific T cell

The nucleocapsid (N) protein is the most common CD4+ and CD8+ T cell target after SARS-CoV-2 infection (65, 74) which dominate the response magnitude, breadth and frequency (66), and polyfunctional N-specific CD8+ T cell response significantly associates with milder COVID-19 disease severity (28). Since N is conserved between different SARS-CoV-2 variants, N specific CD4+ T cells may provide broad protections thus important for universal vaccine against SARS-CoV-2 and even SARS-CoV. Tarke et al. and Heide et al. showed N contained the CD4+ epitopes DQB1*06:02 restricted N261-275, DRB1*07:01 restricted N306-320 and DRB1*03:01 restricted N336-350 et al., giving a possibility about N epitope-specific CD4+ T cells can become immunodominant in certain HLA subtypes, however, whether these epitopes of N protein can clonally expand and function in protection need to be further investigated (13, 65). And it is more interesting to study whether N specific T helper can only help anti-N antibody production, or they can help anti-S and neutralizing antibodies generation. The HLA-B*07:02 restricted N105-113 (SPRWYFYYL) epitope appears to be the most immunodominant SARS-CoV-2 CD8+ T cell epitope to date, and no mutations of this epitope have been observed in the variants of concern. The immunodominant B7/N105+ cytotoxic T cell responses exhibited highly functional avidity and antiviral efficacy to SARS-CoV-2 ancestral strain and multiple variants, which is strongly associated with mild COVID-19 disease, and maintained the long-lasting functional capacity up to 6 months after infection (15, 16, 25). This suggests that N105 may be a preferable target for future vaccine design or adoptive immunotherapy (39, 40), especially against novel variants or subvariants that are prone to S protein mutations and neutralizing antibody escape. Two different views are presented for the origin of the immunodominant B7/N105-specific CD8+ T cell responses. Some studies suggest that they are pre-existing immune cells induced by the previous cross-infection with human coronaviruses (HCoVs). This is supported by evidence that SARS-CoV-2-derived N105 has close amino acid homology to multiple HCoVs (25), and N105-specific CD8+ T cells could medicate cross-reactivity between beta-coronaviruses such as OC43 and HKU-1 which is driven by private TCR repertoire with TRBV27 biased use and longer CDR3β loops (19). Another more prevalent view suggested that the high immunodominance of the N105 epitope stems from the high precursor T cell frequency in unexposed individuals rather than from cross-reaction with HCoVs. Nguyen et al. used the ICS assay combined with the pMHC-I tetramer to determine the mean frequency of B7/N105+ CD8+ T cells in the peripheral blood of COVID-19 patients was 6.88×10 ^-4^, while a high precursor frequency of 3.00×10^-5^ was also observed in unexposed individuals (25). Furthermore, analysis of B7/N105+CD8+ T cell phenotypic profiles in different populations revealed that pre-pandemic unexposed individuals exhibited a predominantly naive T (T_naive_, CCR7+CD27+CD45RA+) cells phenotype, in contrast to the central memory T (T_cm_, CCR7+CD27+CD45RA-) cells dominant phenotype in infected patients. These observations highly suggest that the high frequency of B7/N105+ CD8+ T cells is composed of a naive precursor pool, rather than the cross-reactive memory population formed by previous HCoVs exposure. Single-cell TCR sequencing analysis revealed that CD8+ T cells targeting the N105 epitope exhibit a diverse TCRαβ repertoire as well as promiscuous TCRα-TCRβ gene pairing, with no common clonotype among individuals. This supports that T cells with different clonotypes can respond to the N105 epitope, laying the foundation for the high immunodominance hierarchy (19, 25).

The HLA-B*44:03 and HLA-B*40:01 restricted N322-330 (MEVTPSGTWL)-specific T cell response feature and TCR repertoire signature have also been identified and characterized in several recent studies (16, 20). Robust expansion of B44/N322-specific CD8+ T cells elicited by SARS-CoV-2 infection occurred as early as day 7 after symptom onset, in contrast to anti-nucleocapsid-specific IgG which was not detected until 29 days after symptom onset (16). B44/N322-specific memory CD8+ T cells in COVID-19 convalescent individuals exhibit a high median frequency of approximately 3.6×10^-5^, and remain functional capacity of cytokine production (IFN-γ and TNF) and degranulation (CD107a) with a frequency of about 1×10^-5^ after 104 days post COVID-19 symptom onset, while the anti-S protein IgG titer fell below the detection limit after 79 days post symptom onset (16). These observations suggest that CD8+ T cell responses targeting N protein-derived epitopes at least partly have a faster immune response and long-term persistence after SARS-CoV-2 infection. Similar to B7/N105+ CD8+ T cells, T cells targeting B40/N322 bias use the TRBV27 gene fragment, with a difference that B40/N322-specific T cell TCR repertoire exhibit a common TCRβ chain CDR3 motif TRBV27/TRBJ1-4 (20), indicating the selective recruitment of epitope-specific T cells elicited by infection. The immunodominant conserved epitopes B7/N105- and B44/N322-specific CD8+ T cells have been generated from COVID-19 convalescents and utilized to develop adoptive immunotherapy, exhibited robust functional capacity and cytotoxic potential against different virus variants, which may have important clinical value for immunocompromised patients with lethal infectious complications (39). Other studies have also identified an immunodominant epitope N361-369 (KTFPPTEPK) that can induce specific CD8+ T cell responses in 75% of COVID-19 convalescent individuals in multiple global prevalent HLA allele variants including HLA-A11:01, HLA-B44:01, and HLA-A03:01 (20, 21, 35, 41). This epitope is reported to be highly immunogenic and can induce robust anti-SARS-CoV-2 CD8+ T cell responses in a broad HLA population. Consistent with previous studies, an immunodominant epitope could bind to multiple HLA molecules thereby amplifying the potential immunogenicity of the specific epitope region (13). In addition, Rowntree et al. used TAME technology and found that the magnitude of T cell responses against SARS-CoV-2 internal epitopes (A3/N361, B7/N105, B40/N322, A1/ORF1a1637) is significantly higher than viral surface S proteins (A2/S269, A24/S1208) in seroconverted children and adult, which may reflect the differences in immunodominant pattern and immunodominance hierarchy of different SARS-CoV-2 proteome epitopes (20). Through TCR sequencing analysis, Hu et al. identified two common TCR clonotypes presenting N361 antigen specificity, with TCRβ chain CDR3 sequences ASSRAGTGYNEQF and ASSPSVYFEVSGANVLT, respectively. These 2 TCRs have complementary recognition capacity and exhibit high functional avidity against SARS-CoV-2 ancestral strain and multiple variants, which has important clinical significance for current vaccine design against future variants and subvariants (21).

### 4.2 Spike-derived epitope-specific T cell

The SARS-CoV-2 surface dominant antigen Spike is a key component involving viral invasion. Spike-specific CD4+ T cell responses mediate critical anti-SARS-CoV-2 immune effects and associate with anti-RBD antibody titers suggesting that they are also capable of supporting antibody responses (13). However, it is noteworthy that the frequency and magnitude of Spike specific CD8+ T cell responses after natural infection are seem to generally lower than those of nucleocapsid (20), which may be related to the immunogenicity difference of viral antigens. Recently, a wide range of studies have focused on the immunodominant HLA-A*02:01 restricted S269-277 (YLQPRTFLL) epitope-specific CD8+ T cell response feature and TCR repertoire characteristics (16, 17, 20, 25, 42). Detected by direct *in vitro* TAME, the mean frequency of A2/S269-specific CD8+ T cells is approximately 1.65×10^-6^ in healthy pre-pandemic individuals (25), and 1.44×10^-5^ and 1.28×10^-5^ in the COVID-19 acute and convalescent patients, respectively (17), suggesting that A2/S269-specific CD8+ T cells exhibit a certain level of activation and expansion following natural infection, but significantly lower than N protein-derived N105+CD8+ T cell responses (25). The A2/S269+CD8+ T cells expressed multiple cytotoxic granzymes/perforin, indicating their killing capacity and activation status, but the similar level was also found on the total CD8+ T cells (17). Transient activation of A2/S269-specific CD8+ T cells in COVID-19 patients and T_naive_ and stem cell-like memory T (T_scm_, CCR7-CD27+CD45RA+) cells dominant naïve memory phenotypes in convalescents may further reflect the lower immunodominance hierarchy (17). The suboptimal characteristics of A2/S269+ CD8+ T cells may be derived from the low degree of TCRαβ repertoire diversity, manifested as biased use of TRAV12 gene fragments (37, 42), public TRAV/TRAJ pairing and CDR3α loop motif including TRAV12-1/TRAJ43 CVVNXXDMRF motif (X represents any amino acid) and TRAV12-2/TRAJ30 CAVNXDDKIIF motif (25), which also suggests the essential role of TCRα chain in recognizing this epitope. Although the TRBV gene usage is less common, there remains a bias of TRBV7-9 (20, 37), which is quite distinct from the highly diverse TCR repertoires of immunodominant B7/N105. Overall, the suboptimality of S269 is reflected in multiple aspects, including the specific T cell responses frequency, magnitude, biological function, phenotypic characteristics and TCR repertoires feature.

In contrast, HLA-A24:02-restricted S protein-derived epitopes S1208–1216 (QYIKWPWYI) and S448–456 (NYNYLYRLF) appear to have higher immunodominance over A2/S269. Previous studies using TAME readout showed that the detected frequencies of A24/S448+ and A24/S1208+ CD8+ T cells in COVID-19 individuals are about 6.30~7.71×10^-5^, which was significantly higher than that of unexposed controls of 8.44~9.50×10^-6^, suggesting that naive A24/SARS-CoV-2 epitope-specific CD8+ T cells can strongly clone expand about 7.5 folds following COVID-19 (18, 25). Notably, the biological functions and association with COVID-19 disease severity of A24/S1208- A24/S448-specific CD8+ T cells have not been fully investigated, and therefore future research could focus on. HLA stabilization assays and HLA-peptide complex dissociation assays revealed that the high antigen sensitivity of two A24/epitopes originated from different mechanisms (22). The high recognition of A24/S1208-specific CD8+ T cells results from the high binding stability of the epitope peptide S1208-1216 to HLA-A24:02, while the high TCR affinity of A24/S448-specific CD8+ T cells determines their high recognition with epitope S448-456 (22). Previous studies suggest that intrinsic properties of viral epitopes such as MHC binding affinity and TCR recognition capacity have critical impacts on shaping the immunodominance hierarchy (59, 62). Furthermore, there also seems to be a close relationship between epitope-reactive T cells TCRαβ repertoire characteristics and epitope immunodominance and immunoprevalence. Rowntree and colleagues revealed that A24/S448- and A24/S1208-specific CD8+ T cells have completely different TCR repertoire profiles (18). For A24/S448+CD8+ T cells, prominent gene segment usage (TRBV2/TRBJ2-7) and common TCRβ chain CDR3 motif XXXGYEQYF resulted in a lack of TCRαβ plasticity similar to the subdominant A2/S269 epitope TCR repertoire. In contrast, the A24/S1208+CD8+ T cells display a high degree of TCRαβ diversity among COVID-19 patients similar to B7/N105+ T cells, possibly reflecting their higher immunodominance hierarchy. The highly diverse TCRαβ repertoire can provide a wider range for the selection of high affinity clonotypes for pMHC complexes. Thus, CD8+ T cells with high TCR repertoire diversity can potentially produce a broader antiviral immune response to SARS-CoV-2 ancestral strain and variants (25).

A recent study using overlapping peptide pools stimulation and ICS assay showed that CD4+ T cells targeting multiple SARS-CoV-2 proteins produce a stronger immune response than CD8+ T cells and may mediate greater antiviral immune protection (17). These potent antiviral CD4+ T cell responses may be induced by several immunodominant CD4+ T cell epitopes. Wragg et al. recently identified an S protein-derived HLA-DRB1*15:01 restricted immunodominant CD4+ T cell epitope S751-767 (NLLLQYGSFCTQLNRAL) (27). The DR15/S751-specific CD4+ T cells frequency was detected by tetramer technique up to 1.36×10^-4^ in early convalescent patients from mild to moderate COVID-19, which was 34-fold higher compared to uninfected individuals. Although DR15/S751-specific CD4+ T cells gradually declined over time, they remain stable with a half-life of >377 days and maintain a frequency of 3.8×10^-5^ in the late convalescent phase of 365-450 days after symptom onset. This is significantly longer than the half-life of approximately 200 days of total SARS-CoV-2-specific CD4+ T cells reported in previous studies (74), suggesting that some immunodominant epitopes can potentially induce robust and durable CD4+ T cell responses. TCR sequencing analysis showed that the expression of the TRBV gene in DR15/S751+ T cells displayed a skewed bias toward TRVB24-1, TRVB20-1 and TRBV6-1, with a highly public CDR3β motif CSARRGTEAFF, but the usage of TRAV gene was more diverse. Therefore, its TCR specificity seems to be driven by the TCRβ chain rather than the TCRα chain (27). However, due to the limited availability of MHC class II tetramers, there are relatively few studies on SARS-CoV-2 epitope-specific CD4+ T cell responses, so future efforts should focus on elucidating its critical role in antiviral immunity.

### 4.3 Other peptide epitope-specific T cell

The M protein is a prominent antigen among SARS-CoV-2 structural proteins, but studies have noted that the epitopes from the M protein lack the strong binding capacity to HLA molecules. Thus, it is generally agreed that the high immunogenicity of the M protein comes from the highly expressed genome rather than containing high-quality epitopes (13). The HLA-A*24:02 restricted M198-206 is currently reported the most immunodominant membrane-derived SARS-CoV-2 epitope, and A24/M198-specific CD8+ T cell responses can effectively suppress propagation of variants of concern including omicron strain and associate with COVID-19 clinical severity (38), and thus it appears to be a potential favorable target for next-generation vaccines or adoptive immunotherapy. Notably, despite the low abundance of T cell responses toward SARS-CoV-2 accessory and non-structural proteins (13, 75), some of the early expressed viral proteins can still induce robust T cell immune responses (76). Using single epitope peptide stimulation and IFN-γ production assays, Schulien et al. detected reactive CD8+ T cells toward a wide scope of SARS-CoV-2 proteins in 88.5% of COVID-19 individuals, mainly targeting ORF1ab region which may relate to the longer protein fragment length and containing more prominent epitopes (16). Among them, HLA-A*01:01 restricted immunodominant epitope ORF1ab1637-1646 (TTDPSFLGRY)-specific CD8+ T cells magnitude is remarkably high that reaching up to 7%-25% of the total CD8+ T cells after SARS-CoV-2 acute infection, which is significantly higher than T cell responses induced by other protein-derived epitopes such as S269, S1208, and comparable to immunodominant N105, supporting the previous study that internal epitopes induce stronger CD8+ T cell responses (20, 29, 33). Furthermore, A01/ORF1ab1637+ memory CD8+ T cells remain at a considerably high level with functional IFN-γ and TNF production within 5 months after recovery from critical and severe COVID-19 disease (29, 33). The TCR sequencing revealed that A01/ORF1ab 1637-specific CD8+ T cells exhibit high TCR repertoires diversity and bias use of the TRBV27 gene fragment same as the TCRβ chain characteristics of N105-specific T cells (19, 20). However, the specific role of the TRBV27 gene fragment in T cells-mediated immune response toward SARS-CoV-2 remains unknown. Remarkably, the population with a given HLA typing seem to be more inclined to present a certain SARS-CoV-2 antigenic epitope, such as the HLA-A*01:01 molecule presenting ORF1ab1637 and the HLA-B*07:02 allele variant presenting the N105, thus inducing robust CD8+ T cell responses that contribute to viral clearance and control of disease severity. A limited number of studies have also investigated other ORF1ab- and some ORF3a-derived epitope-specific T cell responses (16, 36). Ferretti et al. used an unbiased screening strategy combined with the ELISpot assay and tetramer technique and identified HLA-A*02:01 restricted immunodominant epitopes A02/ORF1ab3886-3894 (KLWAQCVQL) and A02/ORF3a139-147 (LLYDANYFL) from COVID-19 patients PBMC, and observed that patients with severe disease exhibited fewer tetramer+ T cells than those with mild disease (36). TCR sequencing revealed that both epitopes-specific T cells clonotypes enriched for TRAV gene segments between most COVID-19 individuals, while TRBV gene usage is less common, such as TRAV38-2/DV8 for A02/ORF1ab3886-specific TCR clonotypes and TRAV8-1 for A02/ORF3a139-147-reactive TCR clonotypes. This suggests that a specific TCR Vα composition forms a structural feature that can bind with high-affinity peptide-MHC, thereby supporting the recognition of antigenic epitope (36). In addition to incorporating the above epitopes into COVID-19 vaccine development, some epitope-specific T cells have the potential to be used in TCR-engineered T cell immunotherapy. For example, immunodominant SARS-CoV-2 CD8+ HLA-A*03:01 restricted ORF1 808-816 and HLA-A*01:01 restricted ORF3a 207-217 epitopes were recently found contain highly functional and cytotoxic TCRs and mediating direct killing virus-infected cells (34). Therefore, the information on the epitope-specific T cell TCR repertoire summarized in this review may be of significant value for future novel interventions toward SARS-CoV-2.

## 5 SARS-CoV-2 epitope-specific T cell following vaccination

Over the past three years, several COVID-19 vaccines have been approved for emergency usage by the WHO and were broadly vaccinated worldwide, proven to significantly reduce the hospitalization risk and mortality, dramatically changed vaccination strategies in response to COVID-19 pandemics (43, 44, 77). These vaccines can effectively induce the production of anti-S protein antibodies and poly-specific T cell responses (78). However, studies noted that different vaccine platforms induce different immune response types and intensity, with the adenovirus-based vaccines can elicit a higher magnitude of S protein-specific T cell responses, while mRNA vaccines produce higher antibody titers (79). Optimized vaccine forms that include multiple identified immunodominant T cell epitopes may benefit populations with poor T cell responses induced by vaccination and those with impaired antibody immunity.

Compared to previous studies on T cell responses toward SARS-CoV-2 overlapping peptide pools, focusing on a single epitope provides a more accurate picture of the breadth, magnitude, kinetics feature and functional capacity of vaccine-induced T cell immunity (14). M. Wragg et al. investigated vaccinees with multiple platforms and found that DR15/S751-specific CD4+ T cells expanded approximately 30-fold after the first vaccination in unexposed individuals and further increased following second and booster dose, and repeated vaccination can selectively expand high-avidity T cell clones (27). Longitudinal analysis of the kinetics following BNT162b2 vaccination revealed that rapid expansion of immunodominant epitope DP04/S167-180 (YVSQPFLM)-specific and DR15/S751-specific CD4+ T cells occur as early as day 7 after primary vaccination and peak at days 7-14 after the second dose, maintaining predominantly effector memory phenotype for at least 200 days (26, 27). mRNA vaccine BNT162b2 can also elicit functionally competent and long-lasting viral epitope-specific CD8+ T cell responses targeting A01/S865, A02/S269, A03/S378, A24/S448 and A24/S1208 with the peak frequency of ~3.6×10^-4^ and maintaining full function for at least 80-120 days (14, 22). These observations suggest that COVID-19 vaccines, at least partially such as BNT162b2, can effectively induce rapid, robust and long-lasting immunodominant S protein epitope-specific CD4+ and CD8+ T cell responses that play a critical anti-COVID-19 role. However, only the low frequency and magnitude of subdominant DR15/S236-specific CD4+ T cell responses were driven by the COVID-19 mRNA vaccine, suggesting that the current vaccination strategy will result in the generation of heterogeneous T cell responses against different viral epitopes (14, 27).

Follicular helper T (Tfh) cells located in the germinal centers of secondary lymphoid tissues are a specific subset of the CD4+ T cell population that specializes in helping B cells and mediating protective antibody production (80). A recent longitudinal follow-up analysis demonstrated that the COVID-19 mRNA vaccine can induce strong, persistent and high TCR clonal abundance of PD-1+CXCR5+ DR15/S167-specific Tfh cell responses in lymph nodes, which maintain at high frequency >170-200 days, and were consistent with long-term survival germinal center B cell responses (26). Furthermore, there is a subpopulation of CD4+ T cells with similar phenotype and functional capacity to Tfh cells in peripheral blood named circulating follicular helper T (cTfh) cells (41), which are also strongly driven following COVID-19 vaccination. Activated DR15/S751-specific cTfh cells are rapidly generated at day 5 after BNT162b2 of convalescent individuals, and subsequently remain in circulation at a resting state for long period (27). The frequency of tetramer+ cTfh memory cells prior to vaccination was positively correlated with neutralizing antibody titers after vaccination, emphasizing the value of previously established Sike epitopes-specific cTfh cells in supporting antibody response recall after re-antigen exposure (27).

Some studies demonstrated that there are many differences in SARS-CoV-2 specific T cell immunity induced by different antigen exposure patterns and orders such as COVID-19 vaccination, natural infection, hybrid immunization or breakthrough infection (14, 73). SARS-CoV-2-specific T cells in COVID-19 convalescent individuals predominantly target non-spike protein epitopes, whereas mRNA vaccinees exhibit a broad spike epitopes-specific T cell response (81). The mRNA vaccination for those who recovered from SARS-CoV-2 infection also led to the main expansion of S-protein-specific T cells, whereas breakthrough infections induce robust non-S-protein-specific T cell responses, suggesting that current vaccine strategies tend to recruit Spike-specific T cell population, whereas breakthrough infections increase the diversity of T cell repertoire (73). However, in the context of the constant mutation in the SARS-CoV-2 genome, especially the emergence of the Omicron variant with a wide scope of mutations in S protein epitopes. This should arouse public vigilance that future vaccine regimens may need to incorporate more SARS-CoV-2 proteomic epitopes, especially for immunodominant epitopes that are highly conserved among mutant strains.

## 6 Heterogenous phenotypic characteristics of SARS-CoV-2 specific T cells

Although quite a lot of studies showed that T cells play important role in COVID-19 recovery, the phenotype and the functionality of SARS-CoV-2 specific T cells are not clear, so far, some studies focus on the phenotype defined by CD137 and CD69 (AIM) for virus specific CD8+ T cells, and CD154 and CD137 for CD4+ (11, 27), some studies sticked to the phenotype defined based on IFN-γ+ and TNF-α (ICS) (15). However, Whether SARS-CoV-2 specific CD8+ T cells defined AIM is containing or overlapped with those defined by ICS is unknown, and which one play more important role is not clearly addressed. although both were proved to combating against COVID-19 infection and can be induced in vaccinees. To some extent, as known for its heterogenicity, whether both can differentiate into similar memory T cells subpopulation is also obscure. Here, we summarize the heterogenicity of virus specific T cell in COVID-19.

Among the memory T subpopulations, T_cm_ preserved long-term specific immune memory to SARS-CoV-2 for rapid future responses (9, 82). T_scm_ is capable for self-renewing and multilineage differentiating to broad spectrum of anti-SARS-CoV-2 memory and effector T cell population (83). Effector memory CD45RA T (T_emra_, CCR7-CD27-CD45RA+) cells are virus-experienced cells that re-express the naïve cell marker CD45RA, which were increased in COVID-19 and correlate with milder disease (84). Many previous studies have performed phenotypic characterization and subsets division at the level of total SARS-CoV-2 specific memory T cells (8, 85), demonstrating that SARS-CoV-2-specific CD4+ memory T cells were predominantly T_cm_ cells, whereas SARS-CoV-2-specific CD8+ memory T cells were mainly T_emra_ cells. However, epitopes-specific memory T cells of the different immunodominance hierarchy toward SARS-CoV-2 also appeared to display convergent phenotypic feature, which are prevalent in both CD4+ and CD8+ T cells and exhibit differences from the total SARS-CoV-2-specific T cells. For example, immunodominant epitope DR15/S751+CD4+ and B7/N105+CD8+ memory T cells have similar phenotype profiles exhibiting a stable T_cm_ phenotype (25, 27, 28), while epitopes with slightly lower immunodominance, such as A24/S448, A24/S1208 and A2/S269+CD8, their specific T cells have similar phenotype patterns, displaying highly diverse memory T cell phenotypes including T_cm_, T_scm_, T_emra_ and T_naive_ (18, 25, 31). In contrast, the subdominant DR15/S236+CD4+ memory T cells population exhibits a more T_naive_ memory phenotype profile in COVID-19 convalescent patients (27). These memory phenotype differences observed in recent studies may associate to the longevity and biological function of epitope-specific T cells, thus affecting their immunodominance hierarchy. Notably, the relationship and potential mechanism still needs to be further validated by large sample sizes cohorts and consistent time point comparison in future, which may be of great value for multidimensional decoding of virus epitopes.

Previous studies have reported a significant correlation between different HLA allelic variants and COVID-19 disease severity and clinical outcome (86, 87), while HLA type also seems to associate with the phenotypic characteristics of restricted epitope-specific T cells (16). Schulien et al. found that HLA-A restricted epitopes- (A01/ORF3a207-215, A01/ORF1ab4163-4172, A02/ORF3a139-147) compared to HLA-B restricted epitopes- (B44/N322-330, B44/ORF1ab3946-3954, B07/N105) specific CD8+ T cell more bias to early differentiation of T_cm_ and effector memory 1 T (T_em1,_ CCR7-CD27+CD45RA-) cells subsets, and are highly expressed with antigen recognition-related markers and low expression of cell differentiation-related markers (16). This may partially explain the effect of HLA allelic variants on the COVID-19 outcome by affecting the phenotypic and functional characteristics of various restricted epitopes-specific T cells, thereby changing the T cell immune responses of specific populations after SARS-CoV-2 infection.

Furthermore, the phenotypic characteristics of SARS-CoV-2 epitope-specific T cells distinctly differed among patients with different disease severity from mild or moderate, to severe and critical disease. W. Nelson et al. using the combinatorial TAME strategy analyzed two S-protein-derived epitopes- (S166-177 and S310-320) and two N-protein-derived epitopes- (N305-316 and N329-340) specific CD4+ T cell responses in COVID-19 patients with HLA-DRB1*07:01 allele, and found that the tetramer+ T cell phenotypes differed between disease severity, with more CXCR3+CCR4- Th1 cells in mildly infected individuals and impaired differentiation and formation of Th1-type CD4+ T cells in severe COVID-19 patients (32). This observation is consistent with previous reports that Th1 cells profile plays a key role in effective viral infection resolution and symptoms control, whereas Th2 and Th17 cells are usually associated with more severely ill, possibly due to the close association of lung immunopathology and ARDS, respectively (8, 88, 89). For acute COVID-19 patients with the critical disease, tetramer+CD8+ T cells lack polyfunctional cytokine production, and exhibit high expression of inhibitory receptors and gene expression profiles that limit T cell re-activation and migration (29). Notably, the expression of inhibitory receptors such as PD-1 is associated with T cell activation and function (31), whereas the co-expression of multiple inhibitory receptors (NKG2A, LAG-3, CD-95, and TIGIT) is the phenotypic marker of T cell dysfunction and exhaustion and correlates with the more severe COVID-19 course (30, 90). However, the distribution and contribution of SARS-CoV-2 specific cTfh cells in mild and severe infections are currently controversial (32, 90).

Multiple studies have observed that the phenotypic characteristics of SARS-CoV-2-specific T cells also exist differences in different age groups (20, 25, 91). Analysis of the ex vivo phenotype profiles of epitopes-specific T cells in seroconvert children and adults infected with SARS-CoV-2 revealed that tetramer+ CD8+ T cells of children mainly display naive phenotypes such as T_scm_ or T_naive_ cells, while adults had a higher frequency of effector memory populations (20). This difference may arise from varying degrees of previous exposure to HCoVs and the resulting antigen-experienced T cell differentiation (91). Moreover, a recent study showed that in response to a specific SARS-CoV-2 epitope such as B7/N105, reactive CD8+ T cells in the elderly often exhibit a highly heterogeneous T_naive_, T_cm_, or T_emra_ phenotype, in contrast to the homogeneous phenotypic profile exhibited by adults, which may associate with T cells in the elderly experienced age-related dysregulated homeostatic proliferation or with previous coronavirus infection (25).

## 7 Cross-reactivity of SARS-CoV-2, variants of concern and HCoVs

Broad cross-reactive T cell responses targeting multiple SARS-CoV-2 structural and non-structural proteins have been reported in more than 50% of unexposed healthy individuals (11, 24, 92–95). These immune responses may be derived from preexisting memory T cells generated by previous HCoVs exposure and can cross-recognize SARS-CoV-2. The six existing HCoVs include α coronaviruses (HCoV-NL63, HCoV-229E) and β coronaviruses (HCoV-OC43, HCoV-HKU1, SARS-CoV, SARS-CoV-2, and MERS-CoV). The highly conserved cross-reactive epitopes in HCoVs and SARS-CoV-2 are shared in T cell immunity due to the presence of amino acid sequence homology (96, 97), and thus induce long-term maintenance of cross-reactive cellular immunity (93). Currently, the function and impact of preexisting cross-reactive T cell responses in COVID-19 remain controversial, and some researchers have pointed out that it may be a double-edged sword (9, 98). The highly conserved immunodominant region S811-831 (KPSKRSFIEDLLFNKVTLADA) in SARS-CoV-2 and multiple HCoVs contains multiple MHC class II-restricted CD4+ T cell epitopes (92, 99, 100). In this peptide region, S816-830 cross-reactive CD4+ T cell responses are efficiently recruited to most SARS-CoV-2-infected individuals and almost all mRNA vaccinees, and positively correlate with S protein-neutralizing antibodies, suggesting that coordinated cross-reactive immune responses may play an important anti-SARS-CoV-2 role (92). Moreover, some viral proteins are essential for the viral replication cycle, such as RNA polymerase and helicase, are often highly conserved among multiple coronaviruses and induce strong cross-reactive T cell responses, which may become potential candidate targets for future vaccine design. Swadling et al. and Le Bert et al. detected specific CD8+ T cell responses targeting nsp7, nsp12 and nsp13 in SARS-COV-2 unexposed individuals and exposed healthcare workers and found that these cross-reactive T cells were rapidly reactivated after virus exposure, producing anti-infective effect and having the potential to induce abortive infection (94, 101). Nesterenko et al. identified the SARS-CoV-2-derived nsp12 epitopes RQLLFVVEV and TMADLVYAL with extensive cross-reaction to HCoVs. Their specific TCRs can target and eliminate cells expressing viral RNA polymerase and associate with reduced disease severity (24). These observations support the previous findings that recent HCoVs infection or higher levels of cross-reactive epitope-specific T memory cells appear to be associated with a better COVID-19 clinical outcome (23, 92, 95). However, several other studies have pointed out that HCoV-specific T cells commonly have only low affinity against SARS-CoV-2 antigens (102, 103). These low-affinity and low-functional cross-reactive T cells prevent the formation of high-affinity T cell responses after primary SARS-CoV-2 infection, thus negatively affecting efficient antiviral immunity (98). In general, recall of preexisting cross-reactive memory T cells can produce a rapid early antiviral immune response, which, although not sufficient to prevent SARS-CoV-2 infection, may modulate COVID-19 disease severity or modulate vaccination responsiveness resulting in a faster or stronger vaccine response.

In the context of continually accruing mutations in the SARS-CoV-2 genome, many emerging viral variants such as Omicron and subvariants, which has been globally prevalent in the last two years, are able to generate immune escapes against the ancestral strain, thus greatly increasing the number of breakthrough infection cases. However, T cell immune responses elicited by prior infection or COVID-19 vaccines are greatly preserved and can cross-recognize variants of concern (VOCs) from Alpha to Omicron (104–107). These T cell immune responses are associated with efficient viral clearance and milder disease severity, which is clearly distinct from the severely impaired SARS-CoV-2-specific B cell responses and neutralizing antibody function (108). Based on HLA polymorphisms, each individual recognizes a group of unique T cell epitopes estimated to be at least 30-40 SARS-CoV-2 antigen epitopes, including approximately 19 CD4+ T cell epitopes and 17 CD8+ T cell epitopes (13). At the population level, roughly 80% of the memory T cells elicited by natural infection or vaccination can cross-react with different SARA-CoV-2 variants, and <30% of the T cell responses are lost when identifying common VOCs (105, 106). Therefore, future new variants or subvariants are unlikely to escape this extensive T cell immunity from the individual and population levels. Previously established memory T cells also have extensive immune coverage and cross-recognition capacity against the Omicron variant, mainly targeting conserved immunodominant epitopes including B7/N105, B40/N322, A3/N361, A24/S1208, DPB4/S167, A24/M198, A1/ORF1a1637, thus playing a critical role in limiting the transmission and pathogenesis of Omicron. However, it is worth noting that some epitopes mutations in the RBD region of the Omicron proteome such as N440K mutation (S751-765) will reduce the binding affinity to HLA molecules (109, 110), and some mutations in the accessory protein regions such as A1708D mutation (ORF1ab1707-1716) and I2230T mutation (ORF1ab2230-2238) will impair the activation of CD8+ T cells (111). Under the influence of multiple mutations in critical epitopes, approximately 21% of individuals showed reduced T cell responses against Omicron (112), suggesting that some individuals target non-conserved epitopes resulting in limited T cell immunity. Thus, the constant evolution of SARS-CoV-2 forced vaccine design to focus on S protein sequences that were unaffected by epitope mutations, or to select more conserved immunodominant epitopes. Predicting future circulating variants is a major challenge for vaccine strategies that would otherwise lead to reduced efficacy of vaccination.

## 8 Prospectives

Albeit we accumulate various related studies into this review, in COVID-19 T cell immune filed, there are still a few questions remain to be answered in future. How the SARS-CoV-2 epitope-specific T cell responses were initiated, especially under different circumstance such as vaccinations, infections, vaccination-infection and infection-vaccination patterns, and how these cells rapidly response after several encountering antigen. And after multiple antigen training, whether those epitope-specific T cell multiple-functionality can be improved, whether the starting of epitope-specific T cell induction are different in mild and severe COVID-19 patients, especially under severe situation, and how antigen cross-dressing play in stimulating T cell response.

After epitope-specific T cell responses generated, it is still not known how those T cells differentiate into memory phase, do they correspondingly differentiated between immunodominant and subdominant epitopes, and did they have similar TCR clonal expansion and have similar functionality characteristics? And what host factors drive the clonal expansion, even divided the T cell response between activation and exhaustion. Notably, what role of these epitope-specific T cell responses in the cytokines storm generation, inhibition in early phase or loosen T activation induced overaction. More importantly, how epitope-specific T cells interact with B cell biology, is especially need to study in future. It will be related to the question whether epitope-specific T cells can be further divided into epitope specific Treg, and as mentioned in previous text, whether N, M or ORF specific CD4+ T cells can help anti-S or neutralizing antibodies generation. Additionally, more studies should be carried on the longevity and location of epitope-specific memory T cells, whether COVID-19 can generated T residential memory in lung tissue, how they keep regeneration, whether they could rapidly response secondary COVID-19 infection should be addressed in the future. Notably, while SARS-CoV-2-specific CD8+ T cell epitopes restricted by multiple predominant HLA-I have been well investigated, CD4+ T cell epitopes specific T cell responses have been understudied which is partly due to the limitations in reagents for class-II multimers. However, considering the critical role of CD4+ T cells in anti-SARS-CoV-2, future studies should pay more attention to this issue and balance this research gap.

Finally, what need for greater vigilance is the immune escape caused by SARS-CoV-2 genome mutation poses a great threat to the current vaccine effectiveness. Most of the immunodominant epitopes covered in this review have been confirmed to be highly conserved, and specific T cell response toward these epitopes can cross-recognize the multiple SARS-CoV-2 variants especially for Omicron (**Table 1**). Therefore, future vaccine regimens could incorporate those critical epitopes to improve the efficacy and longevity. When considering the inclusion of these epitopes in vaccine development, the issue of HLA restriction is indeed a concern, one way to overcome HLA limitations, we can constitute a peptide pools, to cover more different global frequent HLA-restricted SARS-CoV-2 immunodominant epitopes in vaccine design, and then combined with novel nanoparticles-based vaccine delivery, thus inducing a broad immune response that covering wider HLA alleles. Second way to constitute conserved SARS-CoV-2 proteins into viral vector or combining with special adjuvants, which can stimulate strong T cells targeting to SARS-CoV-2 proteins when viral vector-based vaccine replicated *in vivo*. Ideally, it will be great to design a live attenuated SARS-CoV-2 vaccine therefore contains all viral proteins, which contains all T cell epitopes and no matter HLA subtypes.

## Author contributions

ZW and JW contributed to the conception and design of this review article. GY wrote the manuscript. PS, JQ, and DC searched and collected the latest literature. NZ, ZW, YZ, and XY critically reviewed the manuscript. All authors contributed to the article and approved the submitted version.
